# Therapeutic Potential of Bioactive Compounds from Traditional Chinese Medicine in Modulating Macrophage Cholesterol Metabolism for Atherosclerosis Treatment

**DOI:** 10.3390/ph18081113

**Published:** 2025-07-25

**Authors:** Lijiao Yan, Jiageng Guo, Dan Huang, Fan Zhang, Zhengcai Du, Xiaotao Hou, Jiagang Deng, Yan Xie, Erwei Hao

**Affiliations:** 1 Guangxi Key Laboratory of Efficacy Study on Chinese Materia Medica, Guangxi University of Chinese Medicine, Nanning 530000, China; as1813572024@163.com (L.Y.); triumph325@163.com (J.G.); dd88hd@126.com (D.H.); zhangfanzf78@126.com (F.Z.); duzhengcai8@163.com (Z.D.); xthou@126.com (X.H.); dengjg53@126.com (J.D.); 2 University Engineering Research Center of Reutilization of Traditional Chinese Medicine Resources, Guangxi University of Chinese Medicine, Nanning 530000, China; 3 Guangxi Key Laboratory of TCM Formulas Theory and Transformation for Damp Diseases, Guangxi University of Chinese Medicine, Nanning 530000, China

**Keywords:** atherosclerosis, macrophage cholesterol metabolism, traditional Chinese medicine, anti-atherosclerosis compounds, mechanism of action

## Abstract

Atherosclerosis (AS) is a complex pathological process characterized by the pivotal involvement of foam cells in its pathogenesis. As the primary cellular components of arterial plaques, foam cells critically determine plaque stability. Foam cells derive mainly from macrophages, and their formation is driven by dysregulated lipid metabolism within these immune cells. Macrophage cholesterol metabolism is a highly regulated process comprising four key phases: uptake, esterification, hydrolysis, and efflux. Under physiological conditions, these four phases maintain a delicate balance. However, disruption of cholesterol homeostasis results in the excessive accumulation of intracellular lipid, promoting the formation of foam cell and inflammasome activation, thereby accelerating the atherosclerotic progression. Therefore, targeting macrophage cholesterol metabolism has emerged as a promising therapeutic approach for AS. This review summarizes the mechanisms underlying macrophage cholesterol metabolism and highlights recent progress in identifying bioactive components of traditional Chinese medicines (TCMs) that mitigate AS through the modulation of macrophage cholesterol homeostasis. These findings may offer novel insights into the development of clinically effective therapies for the prevention of AS.

## 1. Introduction

Cardiovascular diseases (CVDs) are a leading global health burden [1], responsible for approximately one-third of all deaths worldwide according to the World Health Organization [2]. Atherosclerosis (AS), a major underlying cause of CVDs [3], can progress to thrombotic vascular occlusion, leading to life-threatening complications such as stroke and myocardial infarction [4]. AS, with its complex pathogenesis is characterized [5] by the aberrant accumulation of cholesterol within the arterial intima, which leads to the lipid deposition, the proliferation of fibrous tissue, and the formation of atheromatous plaques. These plaques narrow the arterial lumen to cause blood flow problems [6]. Since the late 1970s, macrophages have been recognized as key regulators of the formation of atherosclerotic plaque [7]. Dysfunctional macrophages undergo phenotypic transformation into foam cells, which is a major component of arterial plaques [8,9]. Foam cell formation not only disrupts lipid homeostasis but also triggers multiple cell death pathways, including apoptosis, pyroptosis, and necroptosis. Subsequently, dead cells accumulate to form a necrotic core, which ultimately destabilizes atherosclerotic plaques and exacerbates disease progression [10].

Dysregulated lipid metabolism in macrophages is a key driver of foam cell formation. Macrophage cholesterol metabolism is a tightly regulated process involving four critical phases, including cholesterol uptake, esterification, hydrolysis, and cholesterol efflux [11]. Each is essential for maintaining intracellular cholesterol homeostasis. Under physiological conditions, macrophage cholesterol metabolism remains in dynamic equilibrium. However, elevated extracellular lipid levels can disrupt intracellular metabolic balance and alter the expression of macrophage receptors and lipid-metabolizing enzymes. These dysregulated factors represent promising therapeutic targets for AS.

Currently, the first-line clinical strategy for AS treatment focuses on lipid metabolism regulation, primarily through statin therapy [12]. However, the limitations of statins, including the necessity for long-term administration, adverse effects (e.g., hepatotoxicity, nephrotoxicity), residual cardiovascular risk, and poor patient adherence due to side effects [13,14,15], highlight the urgent need for alternative therapeutic approaches. Traditional Chinese medicines (TCMs), mainly Chinese herbal medicines, have a long history in AS treatment in China, suggesting their potential safety [16,17]. TCMs, characterized by multi-pathways, multi-levels, and multi-targets, have shown promising potential in the prevention and treatment of CVDs [18,19]. Growing evidence indicates that TCMs can attenuate macrophage-mediated lipid metabolism, inflammatory responses to mitigate AS progression, and numerous TCM compounds can modulate macrophage cholesterol metabolism [17,20]. For instance, Qing-Xue-Xiao-Zhi formula, an approved TCM prescription for clinical treatment of AS patients, promotes lipid efflux and inhibits macrophage-mediated inflammation [21]. Alisma Decoction has long been used for treating cardiovascular and cerebral diseases in Clinic. It blocks lipid deposition and inhibits inflammatory response through the activation of the LXRα pathway [22]. Tanshinone IIA from *Salvia miltiorrhiza* suppresses foam cell formation in THP-1-derived macrophages by enhancing cholesterol efflux, and baicalin from *Scutellaria baicalensis* enhances cholesterol efflux and reduces systemic deposition. These findings suggest that targeting macrophage cholesterol metabolism may offer promising strategies for AS management and CVD prevention. The characteristic or major compounds from TCMs that target the macrophage cholesterol metabolism may be potential anti-AS lead compounds. Therefore, the investigation of TCM-derived compounds on the macrophage cholesterol metabolism may provide valuable insights into future drug development for treating AS.

To the best of our knowledge, there is currently a rare comprehensive review of the influence of macrophages’ cholesterol metabolism in AS progression and TCMs’ compounds interfering with the metabolism. Therefore, this review aimed to search for the key regulators in macrophage cholesterol metabolism and the bioactive compounds from TCMs for AS treatment through modulating macrophage cholesterol metabolism. Following meta-analysis procedure [23], Web of Science, Google Scholar, SciFinder, X-MOL, Baidu Scholar, PubMed, and CNKI were used for data collection. These searches were conducted through 2025, with all data searched using the characters “atherosclerosis”, “macrophage”, “foam cell”, “traditional Chinese medicine”, “Cholesterol Metabolism”, “anti-atherosclerosis compounds”, and “Pharmacology”. The search was not limited by language. The searched literature was manually screened to identify the desired content.

This review was conducted firstly to elucidate the macrophages’ dysfunction in cholesterol metabolism and summarize the involved key regulators, and then to present the pharmacological effects and underlying mechanisms of TCMs’ compounds classified by their chemical compositions. By systematically reviewing the recent literature, we aimed to provide the foundation for in-depth research of TCMs’ compounds on the anti-AS effects in clinical practice, identify the research gap, and provide the research directions for TCMs’ compounds in the future.

## 2. The Role of Macrophages in AS

**Figure 1:**
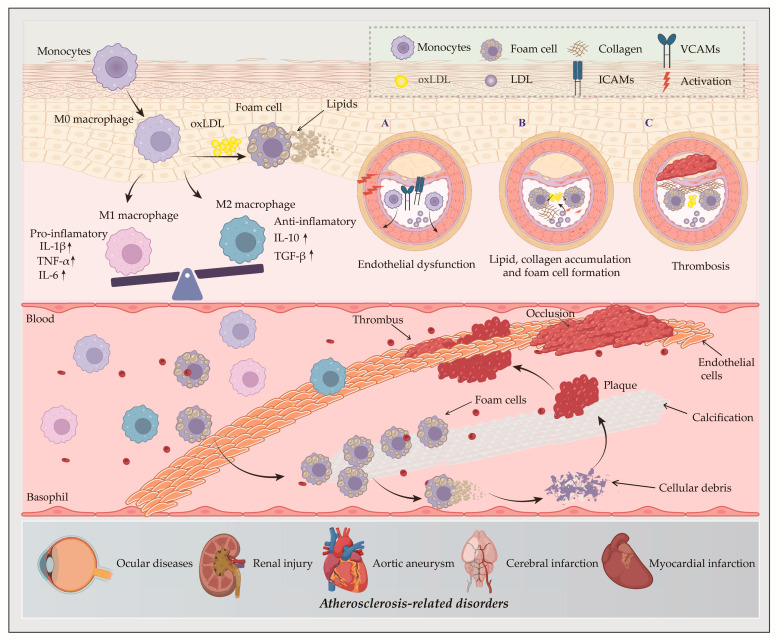
The role of macrophages in AS (“↑” indicates an upward adjustment). Within the micro-environment, monocytes differentiate into macrophages. Normally, these macrophages undergo dynamic polarization from the resting M0 state into M1 (actively secrete pro-inflammatory cytokines: IL-1β, TNF-α, and IL-6) and M2 (actively secrete anti-inflammatory cytokines: IL-10, TGF-β), which are in an equilibrium. Under pathological conditions, macrophages internalize ox-LDL and lipids, leading to foam cell formation. The apoptosis of foam cells results in the formation of necrotic cores, generating unstable plaques. Meanwhile, apoptotic foam cells form calcified micro-vesicles, which act as initiation points for calcification and contribute to plaque rupture. Plaque rupture triggers thrombosis, potentially causing acute vascular occlusion and subsequent cardiovascular events, such as ocular diseases, renal injury, aortic aneurysm, cerebral infarction, and myocardial infarction. (**A**) During early AS, EC dysfunction occurs due to lipid accumulation in the vascular intima. Activated ECs upregulate chemokine secretion and the expression of adhesion molecules, thereby promoting monocyte recruitment and adhesion. (**B**) Macrophages recognize and devour large amounts of ox-LDL and lipids, leading to intracellular lipid and collagen accumulation, the formation of foam cells and the migration of vascular smooth muscle. (**C**) Foam cells accumulate in the arterial intima. The apoptosis of foam cells generates unstable plaques, triggering thrombosis.

Macrophages, which are highly plastic and functionally versatile immune cells, constitute the predominant leukocyte population within AS plaques and play a pivotal role in disease progression (Figure 1).

Within the microenvironment, these monocytes differentiate into macrophages [24]. Normally, these cells undergo dynamic polarization from the resting M0 state into distinct functional phenotypes in response to microenvironmental cues. The two major polarization states include classically activated (M1) macrophages, characterized by secretion of pro-inflammatory cytokines (IL-1β, TNF-α, IL-6) that mediate immune surveillance and the clearance of pathogens, and alternatively activated (M2) macrophages, defined by production of anti-inflammatory factors (IL-10, TGF-β) that regulate immune homeostasis and promote tissue remodeling [25]. Macrophage polarization states and associated functional properties critically regulate atherosclerotic plaque stability, progression dynamics, and ultimate lesion size.

Macrophages serve as the main source of foam cells [26]. During early AS, endothelial cell (EC) dysfunction occurs due to lipid accumulation in the vascular intima [27]. Activated ECs upregulate chemokine secretion and adhesion molecule expression, such as intercellular cell adhesion molecules (ICAMs) and vascular cell adhesion molecules (VCAMs), thereby promoting monocyte recruitment and adhesion. Through surface scavenger receptors (SRs), macrophages internalize oxidized low-density lipoprotein (ox-LDL) and lipids, leading to intracellular lipid and collagen accumulation, the formation of foam cells and the migration of vascular smooth muscle cells. These processes are critical hallmarks of early atherosclerotic lesions [28,29]. Foam cells penetrate the endothelial barrier and accumulate in the arterial intima media in response to the pro-inflammatory activation of ECs. As the AS progresses, foam cell apoptosis contributes to necrotic core formation, generating unstable plaques [30]. Meanwhile, apoptotic macrophages form calcified microvesicles, which act as initiation points for calcification and contribute to plaque rupture. Plaque rupture triggers thrombosis, potentially causing acute vascular occlusion and subsequent cardiovascular events, such as ocular diseases, renal injury, aortic aneurysm, cerebral infarction, and myocardial infarction [31]. As both structural components of plaques and key inflammatory mediators, macrophages participate in all stages of the AS pathogenesis, from initiation to complication [32].

The pathological accumulation of cholesterol in macrophages not only drives foam cell formation but also activates inflammatory pathways, thereby exacerbating atherosclerotic progression. Therefore, therapeutic strategies that target macrophage cholesterol homeostasis by inhibiting foam cell formation and suppressing associated inflammatory responses represent a cornerstone for the prevention and treatment of AS.

## 3. Key Regulators of Macrophage in Cholesterol Metabolism

**Figure 2:**
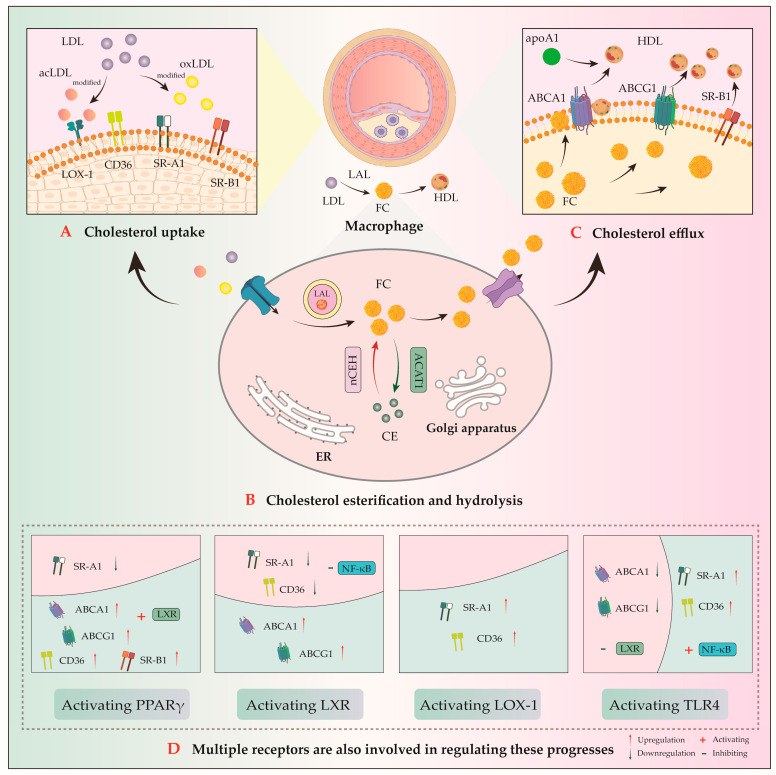
The macrophage cholesterol metabolism and key related receptors. The processes of foam cell formation and macrophage cholesterol metabolism involve cholesterol uptake (**A**), cholesterol esterification and hydrolysis (**B**), and cholesterol efflux (**C**). Multiple receptors are also involved in regulating these processes (**D**). (**A**) The increase in the expression of receptors associated with cholesterol uptake, such as CD36, SR-A1, and SR-B1, leads to rapid recognition and phagocytosis of large amounts of ox-LDL by macrophages. (**B**) In cholesterol esterification and hydrolysis processes, the increase in ACAT expression and the inhibition of the expression of nCEH lead to the accumulation of FC-transformed CE. (**C**) In macrophage cholesterol efflux process, the inhibition of the expression of receptors associated with cholesterol efflux, such as ABCA1, ABCG1, and SR-B1, blocks the efflux of FC. (**D**) PPARγ activation could increase the expression of CD36, ABCA1, ABCG1, and SR-B1, activate LXR while downregulating the expression of SR-A1; LXR activation could increase the expression of ABCA1 and ABCG1 while downregulating the expression of SR-A1 and CD36 and inhibiting NF-κB; LOX-1 activation could increase the expression of SR-A1 and CD36; TLR4 activation could increase the expression of SR-A1 and CD36, activate NF-κB while downregulating the expression of ABCA1 and ABCG1, and inhibit LXR.

Macrophages express multiple transmembrane proteins, which are key regulators of lipid homeostasis (Figure 2). These include the following:

(1) Uptake receptors: scavenger receptor class A1 (SR-A1) and cluster of differentiation 36 (CD36);
(2) Efflux transporters: ATP-binding cassette transporter A1/G1 (ABCA1/ABCG1) and scavenger receptor class B type 1 (SR-B1) [33]. ABCA1-mediated cholesterol efflux serves as the rate-limiting step in reverse cholesterol transport (RCT) process. This process facilitates the transfer of intracellular cholesterol and phospholipids to apolipoprotein A-1 (apoA-1) to generate nascent pre-β high-density lipoprotein (HDL) particles. The RCT pathway ultimately promotes hepatic excretion of excess cholesterol via bile and feces, thereby attenuating AS development [34];
(3) Cholesteryl esterase and hydrolase, cholesterol acyltransferase 1 (ACAT1), and neutral cholesteryl ester hydrolase (nCEH).

As well as macrophage, cholesterol metabolism is regulated by multiple receptors including peroxisome proliferator-activated receptor γ (PPARγ), Liver X receptor (LXR), Lectin-like oxidized low-density lipoprotein receptor-1 (LOX-1), and Toll-like receptor 4 (TLR4).

### 3.1. Cholesterol Uptake

Macrophages primarily uptake extracellular cholesterol through two mechanisms: fluid-phase pinocytosis (cytosolic uptake) and SR-mediated endocytosis. Under physiological conditions, macrophages regulate cholesterol homeostasis by expressing low-density lipoprotein receptors (LDLRs) and SRs on their surface. However, under pathological hypercholesterolemic conditions, elevated extracellular cholesterol levels downregulate LDLR expression, forcing modified lipoproteins such as oxidized LDL to enter macrophages through oxidative modification pathways. Recent studies have identified a novel small-molecule inhibitor, such as Proprotein convertase subtilisin/kexin type 9, that binds to LDLR in the liver and triggers the degradation of LDLR via the lysosomal pathway and maintains cholesterol homeostasis in the body, to be an innovative pharmacological target for treating hypercholesterolemia and AS [35].

#### 3.1.1. Fluid-Phase Pinocytosis

Macrophages mediate phagocytosis of macromolecules through cytosolic processing pathways [36]. Multiple regulatory factors that modulate these cytosolic pathways have been identified, including growth factors, chemokines, interferon-γ (IFN-γ), transforming growth factor-β (TGF-β), and interleukins (IL-33 and IL-17A) [37,38]. Recent in vitro studies demonstrated that cytarabine could enhance LDL and oxLDL uptake in three macrophage models, such as human peripheral blood-derived macrophages, phorbol 12-myristate 13-acetate (PMA)-induced macrophages and macrophage colony-stimulating factor (M-CSF)-differentiated macrophages [39]. Furthermore, minimally oxidized LDL (mmLDL) activates both TLR4 and spleen tyrosine kinase (SYK) signaling pathways in macrophages. This activation triggers cytoskeletal reorganization, enhancing the cellular uptake of both native LDL and oxLDL, thereby accelerating AS progression [40].

#### 3.1.2. SR-Mediated Cytophagy

In addition to fluid-phase uptake (macropinocytosis), macrophages can internalize macromolecules through receptor-mediated endocytosis. The major SRs involved in this process are SR-A1, SR-A2, CD36, and SR-B1. These SRs facilitate lipoprotein internalization by binding to modified LDL such as acetylated LDL (acLDL) and oxLDL [41]. Among all these receptors, SR-A1 and CD36 account for 75–90% of cellular acetylated LDL (acLDL) and oxLDL uptake [42]. Importantly, the uptake of oxLDL mediated by SR-A1 and CD36 is independent of the negative feedback regulation of intracellular cholesterol. This unregulated uptake mechanism is not affected by intracellular cholesterol, resulting in excessive lipid accumulation and free cholesterol (FC) deposition within the vascular wall to accelerate the progression of AS. Studies in *ApoE^−/^^−^* mice have demonstrated that the downregulation of CD36 and SR-A1 expression can reduce macrophage lipid accumulation, thereby inhibiting foam cell formation and attenuating AS development [43].

##### SR-A1

SR-A1, a type II glycoprotein abundantly expressed in macrophages and widely distributed in vascular smooth muscle cells and ECs, functions as a pattern recognition receptor that binds modified LDL particles to facilitate their cellular uptake. Studies have demonstrated that the overexpression of SR-A1 can promote the transformation from macrophage to foam cell [44]. Key evidence from genetic models reveals that *SR-A1^−/^^−^ApoE^−/^^−^* mice exhibit significantly elevated plasma cholesterol levels and reduced atherosclerotic plaque area compared with *ApoE^−/^^−^* controls. Tilianin, a compound from *Dracocepludum moldavica*, has been shown to ameliorate foam cell formation from ox-LDL-induced macrophages by suppressing the cholesterol uptake mediated by SR-A1 [45]. These findings indicate that SR-A1 deficiency could result in reducing intracellular lipid accumulation in macrophages, thereby attenuating foam cell formation and slowing AS progression.

##### CD36

CD36, a class B SR family member, functions as a transmembrane glycoprotein receptor involved in diverse pathophysiological processes, including lipid metabolism [46,47] and immune regulation [48]. In macrophages, CD36 mediates oxLDL uptake through a non-regulated pathway lacking negative feedback control of lipid metabolism [49]. This unregulated uptake leads to excessive intracellular lipid accumulation, ultimately driving foam cell formation [50]. CD36-mediated oxLDL internalization activates PPARγ to create a positive feedback loop that further upregulates CD36 expression and accelerates foam cell formation [51]. This pathway is exacerbated under hyperglycemic conditions, as demonstrated in mouse models where high glucose feeding increased PPARγ levels and CD36 expression in renal cells, resulting in enhanced free fatty acid uptake and intracellular lipid deposition [52]. CD36 also contributes to atherosclerotic inflammation through upregulating pro-inflammatory factors (NF-κB, IL-6, TNF-α, and IL-1β). In addition, pro-inflammatory factors IL-34 mediates CD36 overexpression and promotes foam cell formation through the activation of the p38 mitogen-activated protein kinase (MAPK) signaling pathway [53]. Elevated CD36 mRNA was found in hyperlipidemia patients’ peripheral blood mononuclear cells [54]. The CD36 expression increased in coronary artery disease patients’ monocytes, and statin treatment reduced soluble CD36 and oxLDL uptake [42]. These findings indicate that CD36 is a critical mediator of macrophage-derived foam cell formation in AS progression.

### 3.2. Cholesterol Efflux

RCT represents a pivotal pathway in cholesterol metabolism, serving as the primary endogenous mechanism for cellular cholesterol export and systemic homeostasis maintenance. As the only physiological route for the elimination of excess cholesterol, RCT facilitates the transport of excess lipids from peripheral tissues to the liver for catabolism. Enhanced RCT, particularly through cholesterol efflux mechanisms, has been demonstrated to effectively attenuate AS progression [55,56]. Therefore, RCT has emerged as a promising therapeutic target for anti-atherosclerotic interventions. The critical receptors for the initial step of RCT in atherosclerotic plaques include ABCA1, ABCG1 [57], and SR-B1 [58].

#### 3.2.1. ABCA1

ABCA1, a highly conserved transmembrane protein, serves as a critical mediator of RCT [59]. ABCA1 exerts atheroprotective effects through two primary mechanisms, promoting cellular cholesterol efflux [60] and suppressing inflammatory responses [61]. The functional activity of ABCA1 is closely associated with apoA-1, the major HDL component that facilitates free cholesterol transport. Notably, apoA-1 inhibitors can enhance macrophage RCT by preventing ABCA1 degradation. ABCA1 expression is transcriptionally regulated by the PPARγ-LXRα pathway [62]. Recent studies have identified several TCMs and natural products that modulate this axis. The Ginseng–Honghua–Tongluo formula upregulates ABCA1 expression via PPARγ/LXRα activation, thereby enhancing macrophage cholesterol efflux [63]. Leonurine, a major alkaloid compound isolated from *Leonurus heterophyllus*, could promote cholesterol efflux and alleviate cellular lipid accumulation by magnifying the expression of ABCA1/G1 in a PPARγ/LXRα signaling pathway-dependent manner in human THP-1 macrophage-derived foam cells and abate atherogenesis in *ApoE^−/−^* mice [64]. Active peptides derived from *Hirudinea* inhibited foam cell formation through the LOX-1/LXRα/ABCA1 signaling pathways [65]. In addition, the RNA-binding protein HuR, previously known as riboprobes, was found to increase ABCA1 expression, enhance cholesterol efflux and attenuate foam cell formation by stabilizing ABCA1 mRNA. The deficiency of HuR decreased ox-LDL-induced ABCA1 upregulation [66]. These findings support ABCA1 as a promising therapeutic target for anti-atherosclerotic drug development.

#### 3.2.2. ABCG1

ABCG1, an ATP-binding cassette transporter, plays a crucial role in maintaining tissue and cellular cholesterol homeostasis. This transporter is widely expressed in various cell types [67], with particularly high levels observed in the heart, liver, spleen, lungs, brain, and adrenal glands. Its primary function is to mediate the efflux of intracellular cholesterol to mature HDL, thereby maintaining lipid homeostasis in macrophages. Helal et al. demonstrated that the treatment with extra virgin olive oil upregulated ABCG1 expression and enhanced cholesterol efflux capacity in macrophages [68]. Clinical observations reveal that ABCG1 levels were significantly decreased in patients with coronary heart disease [69]. Experimental evidence from *Ldlr^−/^^−^* mouse models further supports the atheroprotective function of ABCG1. Through inhibiting macrophage-specific ABCG1, the formation of atherosclerotic plaque was exacerbated [70]. Conversely, ABCG1 overexpression enhanced cholesterol efflux, reducing the accumulation of intracellular lipid and attenuating foam cell formation [71]. These findings suggest ABCG1 as a key regulator of macrophage cholesterol homeostasis and a potential therapeutic target for AS management.

#### 3.2.3. SR-B1

The SR-B1 presents different functions in the AS progress. During early AS, SR-B1 promotes macrophage lipid/cholesterol uptake (similar effect as SR-A1) and inhibits ABCA1 activity. As the disease develops, SR-B1 mainly serves as the primary HDL receptor that selectively mediates macrophage cholesterol efflux and hepatic lipid catabolism [72]. Research conducted by Durham et al. demonstrated that atherosclerotic lesions were exacerbated through SR-B1 knockdown in murine models. Particularly, apoA-1 has been shown to attenuate the cardiotoxicity induced by azithromycin via activating SR-B1 [73]. Recent research has challenged conventional paradigms by demonstrating that endothelial SR-B1 actively mediates LDL cellular internalization, rather than passive subendothelial infiltration [74]. The plaque area of advanced atherosclerosis was increased due to the absence of macrophage-specific SR-B1, while decreased by the overexpression of SR-B1 in *ApoE^−/^^−^* mouse models [75]. These findings indicate that SR-B1 is a promising therapeutic target, and agonists of SR-B1 potentially serve as novel agents for coronary AS treatment.

### 3.3. Cholesterol Esterification and Hydrolysis

In macrophages, cholesteryl esters (CEs) and FC maintain a dynamic equilibrium within the cytosol. The extracellular efflux of FC, mediated by membrane receptors, is rate-limited by the hydrolysis of intracellular CEs, as only after hydrolysis can FC be transported across the membrane. When FC efflux is blocked, excess FC leads to excess CEs accumulated as cytoplasmic lipid droplets. Elevated FC levels induce re-esterification in the endoplasmic reticulum, forming CEs to attenuate FC-induced cytotoxicity. This critical balance is regulated by two principal cholesteryl esterases and hydrolases in macrophage, ACAT1 and nCEH.

#### 3.3.1. ACAT1

ACAT1, also termed sterol O-acyltransferase, plays a pivotal role in maintaining cholesterol metabolic homeostasis. It catalyzes the esterification of FC to CEs in the endoplasmic reticulum. Mammals express two ACAT isoforms, ACAT1 and ACAT2, each distributed in distinct tissues. ACAT1 is ubiquitously expressed and is particularly abundant in macrophages, where it serves as the primary enzyme for intracellular CE synthesis [76]. While ACAT2 is expressed in enterocytes and hepatocytes, where it primarily mediates lipoprotein assembly and secretion [77,78]. Foam cells markedly elevated ACAT1 expression [79]. Functional research demonstrated that ACAT1 deficiency induced evaluated FC levels and enhanced cholesterol efflux in mouse peritoneal macrophages, while ACAT1 overexpression promoted CE accumulation [80]. Targeting ACAT1 in *ApoE^−/−^* mice induced atheroprotective effects, such as reduced plasma cholesterol levels and decreased atherosclerotic plaque area [81]. The PPARα and PPARγ pathways are both involved in Cpn-induced macrophage-derived foam cell formation by upregulating SR-A1 and ACAT1 and downregulating ABCA1/G1 expression [82]. These findings indicate that CE synthesis is attenuated by inhibiting ACAT1, thereby slowing AS progression.

#### 3.3.2. nCEH

nCEH is a major macrophage enzyme responsible for hydrolyzing CEs into FC and fatty acids, which is a critical process for intracellular cholesterol efflux. The actions of ACAT1 are opposed to nCEH. nCEH has emerged as a promising therapeutic target for attenuating foam cell formation and AS progression. Macrophages express two distinct CEH isoforms with differential subcellular localization. Research evidence demonstrates that nCEH, also termed hormone-sensitive lipase, was significantly upregulated in both murine peripheral blood macrophages and atherosclerotic lesions. Genetic studies in *ApoE^−/−^* mice reveal that nCEH1 deficiency doubled AS susceptibility compared with wild-type controls. nCEH functions as the key rate-limiting enzyme in cholesterol efflux through hydrolyzing CEs to generate FC [83,84]. This enzymatic conversion is essential since only FC (not CEs) can be transported out of cells. The hydrolysis process determines overall cholesterol efflux capacity. Notably, although nCEH overexpression enhances CE hydrolysis in lipid-laden macrophages, this single intervention provides limited atheroprotection unless accompanied by concomitant ACAT1 downregulation.

Optimal therapeutic efficacy requires balanced modulation between nCEH-mediated hydrolysis (FC generation) and ACAT1-mediated esterification (CE formation).

### 3.4. Cholesterol Metabolism-Related Receptors

Macrophages maintain cholesterol homeostasis through a sophisticated receptor-mediated regulatory network, the dysregulation of which is closely associated with AS. Key regulators include PPARγ and LXRα, which coordinate lipid metabolism by modulating macrophage SR expression and upregulating cholesterol efflux genes (ABCA1/ABCG1); LOX-1, a critical mediator of macrophage cholesterol uptake; and TLRs that recognize mmLDL and promote inflammation and lipid accumulation via the MyD88/NF-κB pathway. Among these regulators, the PPARγ-LXRα-ABCA1 axis represents the most extensively characterized pathway, playing a pivotal role in AS through its regulation of RCT [85].

#### 3.4.1. PPAR

PPAR family comprises three isoforms, each with distinct functions. PPARβ primarily enhances fatty acid β-oxidation in extrahepatic tissues. PPARα regulates hepatic lipid metabolism. PPARγ serves as the master regulator for adipocyte differentiation and lipid storage. High expression levels of PPARγ were observed in plaque macrophages and foam cells. PPARγ presented dual modulation of cholesterol homeostasis through transcriptional activation of cholesterol efflux genes (e.g., ABCA1, ABCG1), suppression of pro-inflammatory factors, and promotion of anti-inflammatory M2 macrophage polarization [86,87]. The activation of PPARγ can increase CD36 expression, whereas it attenuates SR-A1 expression [88]. PPARγ modulates atherosclerotic plaque progression through maintaining macrophage lipid homeostasis and suppressing vascular inflammation.

Experimental studies have demonstrated that several traditional herbal formulations and natural compounds exert atheroprotective effects through PPAR-mediated pathways. In *ApoE^−/−^* mice, dandelion from alcohol extract of *Taraxacum officinale* enhanced macrophage cholesterol efflux via activating the PPARα/ABCA1 pathway, thereby ameliorating hyperlipidemia and associated inflammation [89]. Siwei decoction attenuated high-fat diet-induced hyperlipidemia and reduced atherosclerotic plaque formation in New Zealand rabbits by potentiating the PPAR-LXRα-ABCA1 signaling axis [90]. Ginkgo Tongzhi decoction attenuated AS through coordinated activation of the macrophage PPAR-LXRα-ABCA1/ABCG1 pathway [91]. The active components of *Citrus aurantium* inhibited macrophage foam cell formation by modulating the PPARγ-LXRα-ABCG1/SR-B1 signaling network [92].

#### 3.4.2. LXR

LXR serves as a crucial regulator of cholesterol homeostasis and participates in multiple pathophysiological processes in AS. There are two LXR isoforms, LXRα and LXRβ. LXRα is predominantly expressed in lipid-metabolizing tissues, for example, macrophages, liver, and adipose tissue. In comparison, LXRβ is ubiquitously expressed across all organs. Mechanistic studies demonstrate that the ABCA1 expression is upregulated, cholesterol efflux and RCT enhanced, and lipid accumulation reduced in macrophage foam cells by activating LXR. Conversely, LXR downregulation impairs cholesterol efflux and exacerbates AS. Pharmacological LXR agonists exert atheroprotective [93] effects through inducing expression of LXR target genes (including ABCA1), thereby promoting cholesterol efflux to apoA-1 and HDL and inhibiting foam cell formation and plaque development [94]. 27-hydroxycholesterol, a high-affinity endogenous LXRα ligand, significantly downregulates the expression of macrophage SR-A1, CD36 [95]. These findings demonstrate that LXRα exerts bidirectional regulation of macrophage cholesterol metabolism by simultaneously enhancing cholesterol efflux through ABCA1/ABCG1 upregulation and inhibiting cholesterol uptake via suppressing CD36. This dual regulatory mechanism is essential for maintaining cellular cholesterol homeostasis to attenuate atherosclerotic progression. Moreover, LXR activation inhibits NF-κB-mediated inflammatory responses [96].

#### 3.4.3. LOX-1

LOX-1, a type II transmembrane glycoprotein predominantly expressed in vascular ECs and macrophages, plays a pivotal role in macrophage foam cell formation. The ox-LDL and its hydrolytic products potently induce LOX-1 expression [97]. Under physiological conditions, LOX-1 mediates only 5–10% of total ox-LDL uptake. However, LOX-1 expression is markedly upregulated during inflammatory states. LOX-1 elevates macrophage ox-LDL uptake up to 40% by elevating the expression of SR-A1 and CD36 [98]. This LOX-1-dominated uptake mechanism exacerbates intracellular lipid accumulation and foam cell formation.

#### 3.4.4. TLR4

TLR4, a key pattern recognition receptor expressed in macrophages and vascular ECs (including those in pulmonary tissue), serves dual roles in pathogen recognition through microbial-associated molecular patterns and activation of pro-inflammatory signaling cascades. TLR4 deficiency could markedly attenuate atherosclerotic plaque progression and reduce both lesion size and lipid accumulation. Using TLR4 knockout mice that have been transplanted with bone marrow from LDL receptor knockout mice, it was found that deficiency of TLR4 protects macrophages from lipid accumulation during AS [99]. The potent immunogenicity of ox-LDL is mediated through upregulating TLR. Specifically, oxidized phospholipids within ox-LDL serve as ligands that bind and activate macrophage TLRs. The ox-LDL stimulation significantly enhances TLR2 and TLR4 expressions on macrophage surface, triggering downstream pro-inflammatory cascades including NF-κB and MAPK signaling in atherosclerotic plaques. The activation of these pathways exacerbates local inflammation and promotes M1 macrophage polarization [100]. Notably, the activation of TLR9 induces secretion of multiple inflammatory mediators, including Tumor necrosis factor-α (TNF-α), ICAM-1, and VCAM-1 [101]. Conversely, inhibiting TLR can upregulate cholesterol transporters (ABCA1, ABCG1, SR-B1), resulting in enhancing cholesterol efflux capacity and attenuating foam cell formation [102]. TLR4 activation upregulates the expression of CD36 and SR-A1 through NF-κB-mediated transcriptional regulation [103]. These findings demonstrate that dysregulated TLR signaling plays a central role in macrophage-driven AS.

## 4. Modulation of Cholesterol Metabolism in Macrophages by TCM Components

**Figure 3:**
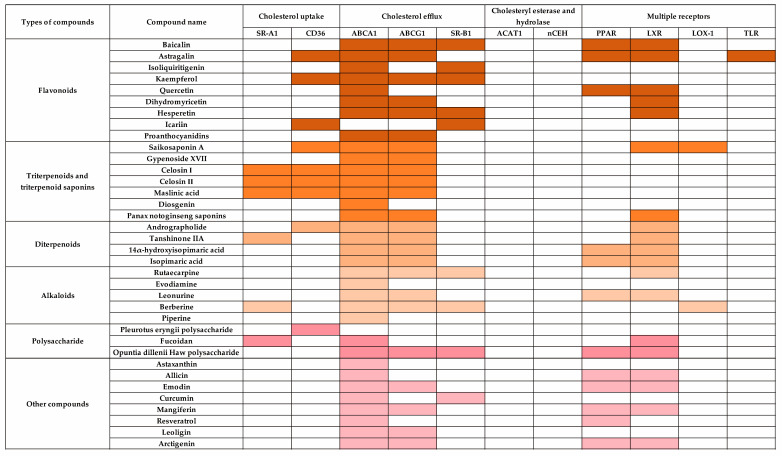
Summary of key regulators influenced by 36 TCM components in macrophage cholesterol metabolism. The color scheme in this figure delineates distinct categories of chemical compounds.

Through searching the databases, we found that 36 compounds from TCMs were found to have effects in modulating macrophage cholesterol metabolism in AS pathogenesis. Among the key regulators involved in macrophage cholesterol metabolism (Figure 3), mainly through the activation of the PPARγ/LXRα signaling pathway, which subsequently enhances the expression of cholesterol transporters (ABCA1 and ABCG1) to modulate macrophage cholesterol metabolism, the underlying mechanism of each compound is presented in the following sections.

### 4.1. Flavonoids

**Figure 4:**
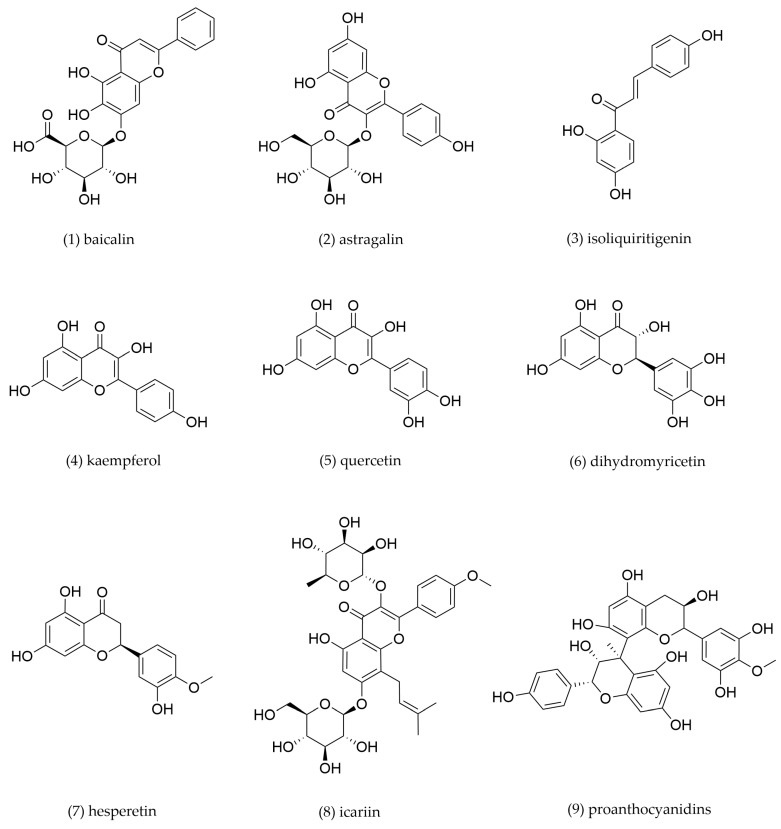
Structure of flavonoids.

Flavonoids, a major class of plant secondary metabolites, are ubiquitously distributed in the plant kingdom. Structurally, flavonoids are divided into subclasses such as flavonoids, isoflavones, flavanols, flavan-3-ols, flavanones, and anthocyanins. In recent years, flavonoids have attracted enormous scientific interest due to their diverse bioactivities and therapeutic potential, including antioxidant, anti-inflammatory, antimicrobial, hypolipidemic, and cardioprotective effects. Flavonoids have been demonstrated with promising efficacy in ameliorating lipid metabolism disorders and mitigating hyperlipidemia-related metabolic diseases. Major constituents from TCM and their underlying mechanisms are presented and summarized in Figure 4 and Table 1.

Baicalin, a major bioactive constituent derived from *Scutellaria baicalensis*, exerts therapeutic effects against hyperlipidemia by acting as a peroxisome PPARγ agonist. Yu et al. found that 50 μM baicalin significantly promoted cholesterol efflux in THP-1 macrophages treated with 50 mg/L oxLDL, and its maximal effect was observed in the treatment beyond 12 h. Western blotting and RT-qPCR analysis further indicated that baicalin treatment upregulated the expression of SR-B1, PPARγ, and LXRα in a dose- and time-dependent manner. Furthermore, pre-treatment with the SR-B1 inhibitor inhibited baicalin-induced cholesterol efflux. It was clarified that baicalin-accelerated cholesterol efflux was mediated by PPARγ/LXRα pathway in THP-1 macrophages by using antagonists and agonists of PPARγ and LXRα [104]. He et al. investigated the therapeutic effects of baicalin in an atherosclerotic rabbit model and explored its mechanism of action in THP-1 macrophages. The study demonstrated that baicalin treatment significantly attenuated atherosclerotic lesion formation and reduced lipid accumulation in the aortic plaques of experimental rabbits. Western blotting analysis revealed that baicalin administration markedly upregulated the protein expression levels of PPARγ, LXRα, ABCA1, and ABCG1 compared with the model control group [105]. These findings suggest that baicalin exerts anti-atherosclerotic effects through activation of the PPARγ/LXRα signaling pathway, which subsequently enhances the expression of cholesterol transporters (SR-B1, ABCA1, and ABCG1) to promote macrophage cholesterol efflux and inhibit the formation of foam cells.

Astragalin, a bioactive flavonoid compound abundant in *Astragalus membranaceus*, exhibits hypolipidemic activities [106]. Zhao et al. demonstrated that astragalin significantly attenuated atherosclerotic lesion progression in *ApoE^−/^^−^* mice. Mechanistic investigation revealed that astragalin activated the PPARγ/LXRα signaling pathway in THP-1-derived macrophages, leading to upregulation of ABCA1 and ABCG1 expression and subsequent enhancement of cholesterol efflux. Furthermore, astragalin treatment downregulated TLR4 expression and suppressed NF-κB nuclear translocation. These findings suggest that astragalin protects against AS possibly by promoting ABCA1 and ABCG1-mediated cholesterol efflux and inhibiting pro-inflammatory mediator release [107].

Isoliquiritigenin, a bioactive chalcone-type flavonoid derived from *Glycyrrhiza uralensis*, exhibits hypolipidemic and anti-atherosclerotic properties [108]. Du et al. demonstrated that treatment of 0.5 μg/mL isoliquiritigenin for 12 h significantly enhanced PPARγ and ABCA1 protein expression while downregulating CD36 expression in peritoneal macrophage-derived foam cells from *ApoE^−/^^−^* mice. Isoliquiritigenin treatment in female *ApoE^−/^^−^* mice decreased the plasma cholesterol levels of very low-density lipoprotein (VLDL)/LDL, promoted plasma superoxide dismutase (SOD) and paraoxonase-1 (PON1) activities, and decreased plasma IL-6, TNF-α, and MCP-1 levels. Moreover, isoliquiritigenin altered the levels of several key genes (such as SR-B1, ABCA1, ABCG8, PPARγ, and fatty acid synthase (FASN)), which were involved in the cholesterol-selective uptake and excretion into bile, triglyceride biosynthesis, and inflammation. The finding suggests that isoliquiritigenin exerts its atheroprotective effects primarily through PPARγ-mediated regulation [109].

Kaempferol is the predominant flavonoid in *Kaempferia galanga*. Recent work by Li et al. demonstrated that kaempferol modulated cholesterol homeostasis in THP-1 macrophages through the downregulation of CD36 expression to suppress ox-LDL uptake and upregulation of ABCA1, SR-B1, and ABCG1 expressions to enhance cholesterol efflux. The c-Jun-AP-1 was identified as the key transcriptional factor involved in the downregulation of CD36 expression induced by kaempferol. The induced expression of ABCA1, SR-B1 and ABCG1 by kaempferol is accompanied by increased heme oxygenase-1 (HO-1) expression. It is suggested that the anti-atherogenic effect of kaempferol is by inhibiting c-Jun-AP-1 and enhancing HO-1 protein [110].

Quercetin, a flavonoid abundant in many TCMs, for example *Sophora japonica* and *Forsythia suspensa*, exhibits significant heart-related benefits. Extensive experimental evidence demonstrates its efficacy in mitigating key pathological processes of AS, including oxidative stress attenuation, anti-inflammatory modulation, and endothelial function enhancement [111,112]. Sun et al. revealed that quercetin upregulated the expression of ABCA1 through activating PPARγ/LXRα in THP-1-derived foam cells, thereby promoting cholesterol efflux and suppressing foam cell formation. Its effect was abolished when the PPARγ activity was inhibited by siRNA knockdown or the addition of chemical PPARγ inhibitor [113].

Dihydromyricetin, a bioactive flavonoid derived from *Ampelopsis megalophylla*, exhibits pleiotropic pharmacological effects including potent antioxidant, anti-inflammatory, and hypolipidemic activities [114,115]. Zeng et al. demonstrated that dihydromyricetin increased mRNA and protein expressions of ABCA1 and ABCG1 in THP-1-derived macrophages. The upregulation of ABCA1 and ABCG1 by dihydromyricetin was dependent on the increased LXRα. The effect of dihydromyricetin on modulating cholesterol homeostasis was through the LXRα-ABCA1/ABCG1 signaling to promote macrophage cholesterol efflux [116].

Hesperetin, a bioactive flavonoid abundant in *Citri reticulatae*, exhibits potent anti-atherosclerotic properties. Sugasawa, N et al. demonstrated that hesperetin significantly lowered plasma total cholesterol level in *ApoE^−/^^−^* atherosclerotic mice. Hesperetin mediated the atheroprotective effects by inhibiting the activity of 3-hydroxy-3-methylglutaryl coenzyme A (HMG-CoA) reductase [117]. Chen et al. found that hesperetin treatment upregulated LXRα expression and its downstream targets (ABCA1, ABCG1, SR-B1 and phosphorylated-AMPK) in THP-1 macrophages. Meanwhile, hesperetin induced increased levels of LXRα expression and its downstream targets, which inhibit the formation of foam cells and promote cholesterol efflux. The hesperetin-induced inhibition of foam cell formation and promotion of cholesterol efflux were decreased by transfection of AMPKα1/α2 siRNA [118].

Icariin, the major active constituent of *Epimedium brevicornu*, has been demonstrated to modulate macrophage lipid metabolism. Yang et al. presented that icariin treatment downregulated CD36 while upregulated SR-B1 expression in a dose-dependent manner in THP-1 macrophages [119]. It was suggested that icariin attenuated ox-LDL uptake and intracellular cholesterol to inhibit foam cell formation by downregulating the expression of CD36 and upregulating the expression of SR-B1. Furthermore, the downregulation of CD36 by icariin was through the p38MAPK pathway.

Proanthocyanidins, a class of polyphenolic flavonoids abundant in TCMs such as *Crataegus altaica*, are composed of flavanol monomers (e.g., catechin and epicatechin) and their oligomeric/polymeric derivatives. These compounds exhibit significant cardioprotective properties against various CVDs. Jamuna et al. demonstrated that impaired autophagy presented in the ox-LDL-induced THP-1 cells was significantly improved by treatment with proanthocyanidins. These effects were achieved by upregulating the expressions of ABCA1 and ABCG1 and promoting cholesterol efflux through Class III PI3K/Beclin1 pathway [120].

**Table 1:** The mechanism of action of flavonoids.

Compound No.	Components	Source TCM	Experimental Model	Dosage	Pathway	Mechanism	Pharmacological Effects	Ref.
**1**	Baicalin	*Scutellaria baicalensis*	THP-1-derived foam cells	2, 10, 50 μM incubation for 48 h	PPARγ/LXRα	SR-B1↑, PPAR-γ↑, LXRα↑	Promote cholesterol efflux	[104]
			THP-1-derived foam cells	25, 50, 100 μM incubation for 24 h	PPARγ/LXRα- ABCA1/ABCG1	PPARγ↑, LXRα↑, ABCA1↑, ABCG1↑	Promote cholesterol efflux	[105]
**2**	Astragalin	*Astragalus membranaceus*	THP-1-derived foam cells	5, 10, 20, 40 μg/mL incubation for 48 h	PPARγ/LXRα	ABCA1↑, ABCG1↑, TLR4↓	Promote cholesterol efflux	[107]
**3**	Isoliquiritigenin	*Glycyrrhiza uralensis*	Peritoneal macrophage-derived foam cells	0.5 μg/mL incubation for 12 h	PPARγ	PPARγ↑, ABCA1↑, CD36↓	Promote cholesterol efflux, inhibit cholesterol intake	[109]
		*Glycyrrhiza uralensis*	*ApoE^−/−^* mice	0, 20, 100 mg/kg/day for 12 weeks	/	VLDL/LDL↓, SOD↑, PON1↑, IL-6↓, TNF-α↓, MCP-1↓, SR-B1↑, ABCA1↑, ABCG8↑, CYP7A1↑ and CYP27A1↑	Promote cholesterol efflux, inhibit cholesterol intake	[109]
**4**	Kaempferol	*Kaempferia galanga*	THP-1-derived foam cells	2.5, 5, 10 μg/mL incubation for 24 h	c-Jun-AP-1/ HO-1	ABCA1↑, ABCG1↑, SR-B1↑, CD36↓	Promote cholesterol efflux, inhibit cholesterol intake	[110]
**5**	Quercetin	*Sophora* *japonica*, *Forsythia* *suspensa*	THP-1-derived foam cells	25, 50, 100, 200 μM incubation for 24 h	PPARγ/LXRα	PPARγ↑, ABCA1↑	Promote cholesterol efflux	[113]
**6**	Dihydromyricetin	*Ampelopsis megalophylla*	THP-1-derived foam cells	1, 10, 100 μM incubation for 24 h	LXRα/ABCA1/ ABCG1	LXRα↑, ABCA1↑, ABCG1↑	Promote cholesterol efflux	[116]
**7**	Hesperetin	*Citri* *reticulatae*	THP-1-derived foam cells	10, 50, 100 μM incubation for 24 h	LXRα/AMPK	LXRα↑, ABCA1↑, ABCG1↑, SR-B1↑, phosphorylated- AMPK↑	Promote cholesterol efflux	[118]
**8**	Icariin	*Epimedium brevicornu*	THP-1-derived foam cells	0.8, 4, 20 μM incubation for 12 h	p38MAPK	SR-B1↑, CD36↓, p38 MAPK↓	Promote cholesterol efflux, inhibit cholesterol intake	[119]
**9**	Proanthocyanidins	*Crataegus altaica*	THP-1-derived foam cells	100 μg/mL incubation for 72 h	Class III PI3K/Beclin1	ABCA1↑, ABCG1↑	Activate autophagy, promote cholesterol efflux	[120]

“↑” indicates an upward adjustment, “↓” indicates downward adjustment.

### 4.2. Triterpenoids and Triterpenoid Saponins

**Figure 5:**
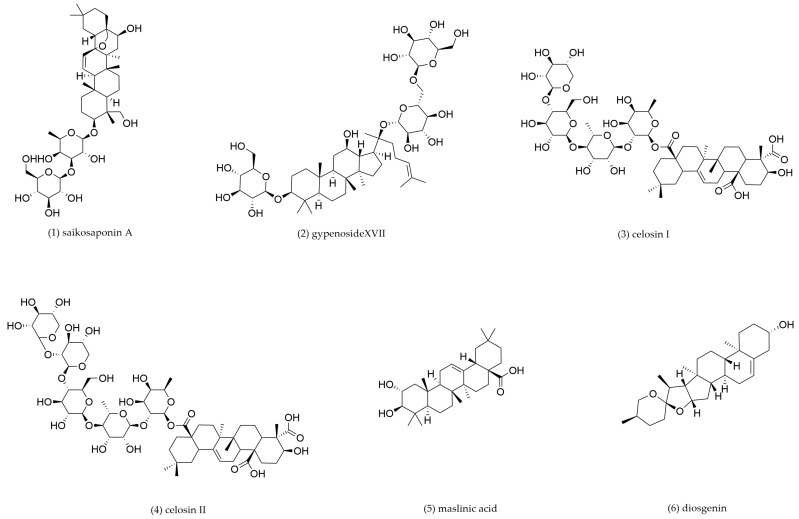
Structure of triterpenoids and triterpenoid saponins.

Triterpenoids and triterpenoid saponins, which are widely distributed in terrestrial plants, have been demonstrated with promising efficacy in lipid lowering and regulating the metabolism of macrophages. Major constituents from TCM and their underlying mechanisms are presented and summarized in Figure 5 and Table 2.

Saikosaponin A, a triterpenoid saponin derived from *Bupleurum chinense* and *Bupeurum scorzonerifolium*, serves as a primary bioactive compound with demonstrated pharmacological properties, including anti-inflammatory activity [121] and anti-atherosclerotic activities [122]. Using primary BALB/c mouse macrophage-derived foam cells, Wei et al. revealed that saikosaponin A treatment upregulated the expression of LXRα, ABCA1, and ABCG1. The formation of lipid rafts was disrupted by depleting cholesterol and inhibiting TLR4 translocation into lipid rafts through activating LXRα-dependent cholesterol efflux pathway [123]. He et al. showed that the anti-atherosclerotic mechanism of saikosaponin A in THP-1 cells was achieved through dual regulation of cholesterol homeostasis, upregulating ABCA1 and PPARγ while suppressing LOX-1 and CD36 expression [124].

Gypenoside XVII, a ginsenoside monomer derived from *Gynostemma pentaphyllum*, has been widely investigated for its potential in CVD prevention. Yang et al. demonstrated that Gypenoside XVII significantly decreased blood lipid levels and atherosclerotic lesion size in *ApoE^−/^^−^* mice. Gypenoside XVII significantly prevented ox-LDL-induced endothelial injury by increasing anti-apoptotic proteins and antioxidant protein expression through the ERα-mediated PI3K/Akt pathway [125]. Deng et al. revealed that Gypenoside XVII (100 μg/mL) upregulated the expression of ABCA1, ABCG1, and miR-182–5p while suppressing histone deacetylase 9 (HDAC9) in THP-1-derived macrophages. In addition, Gypenoside XVII promoted the M2 phenotype of the macrophage. These findings suggest that the protective effect of Gypenoside XVII is achieved via activating the miR-182–5p/HDAC9 signaling pathway, as the over-expression of HDAC9 or suppression of miR-182–5p eliminates the effects of ABCA1/G1 expression, lipid deposition, and pro-inflammatory response [126].

Celosins, a class of triterpenoid saponins derived from *Celosia argentea*, exhibit hepatoprotective, lipid-lowering, and anti-inflammatory activities. Tang et al. reported that celosin administration (10.0–90.0 mg/kg) significantly attenuated dyslipidemia in atherosclerotic mice. Further mechanistic investigation using primary peritoneal macrophages isolated from C57BL/6J mice revealed that the anti-AS effect of celosins may be related to its promoting autophagy. Celosia argentea saponins I and II markedly suppressed macrophage lipid uptake and foam cell formation. This anti-atherogenic effect is achieved through dual modulation of SRs and cholesterol transporters: downregulation of CD36 and SR-A1 expression and upregulation of ABCA1 and ABCG1 [127].

Maslinic acid, a pentacyclic triterpenoid compound derived from *Crataegus pinnatifida*, exhibits multiple pharmacological properties including cardioprotective effects [128]. Mechanistic research demonstrated that maslinic acid exerted both preventive and therapeutic effects against AS through three key pathways: inhibition of NF-κB-mediated inflammatory signaling, downregulation of SRs (SR-A1 and CD36) [129], and upregulation of cholesterol transporters ABCA1 and ABCG1 [130].

Diosgenin, a steroidal saponin derived from *Dioscorea polystachya*, has been demonstrated to lower lipid levels in high-fat diet-fed rats through accelerating RCT and enhancing cholesterol elimination [131]. Lv et al. found that diosgenin significantly upregulated ABCA1 expression in foam cells derived from human THP-1 macrophages and mouse peritoneal macrophages. The underlying mechanism is suggested to be related to miR-19b. Through suppressing macrophage miR-19b expression, diosgenin enhanced ABCA1-dependent cholesterol efflux and inhibited aortic AS progression [132].

*Panax notoginseng* saponins are the primary active components of *Panax notoginseng*. To date, over 80 distinct saponins have been isolated from *Panax notoginseng*, with notoginsenoside R1, ginsenosides Rg1, Re, Rb1, and Rd as the most well investigated. These compounds are widely utilized in the treatment of cardiovascular and cerebrovascular diseases [133]. *Panax notoginseng* saponins have been demonstrated to have significant anti-atherosclerotic activities [134]. Research by Fan et al. indicated that total saponins from *Panax notoginseng* enhanced LXRα expression and subsequently upregulated the cholesterol transporters ABCA1 and ABCG1 in THP-1-derived cells [135].

**Table 2:** The mechanism of action of triterpenoids and triterpenoid saponins.

Compound No.	Components	Source TCM	Experimental Model	Dosage	Pathway	Mechanism	Pharmacological Effects	Ref.
**1**	Saikosaponin A	*Bupleurum chinense*, *Bupeurum* *scorzonerifolium*	C57/BL6J mouse peritoneal macrophage-derived foam cells	3, 6, 12 μM incubation for 12 h	LXRα	LXRα↑, ABCA1↑, ABCG1↑	Promote cholesterol efflux	[123]
			THP-1-derived foam cells	6.25, 12.5, 25, 50 μM incubation for 24 h	PPARγ/LOX-1	PPARγ↑, ABCA1↑, CD36↓, LOX-1↓	Promote cholesterol efflux, inhibit cholesterol intake	[124]
**2**	Gypenoside XVII	*Gynostemma pentaphyllum*	THP-1-derived foam cells	100 μg/mL incubation for 24 h	miR-182-5p/HDAC9	ABCA1↑, ABCG1↑, miR-182–5p↑, HDAC9↓	Promote cholesterol efflux	[126]
**3**	Celosin I	*Celosia argentea*	C57/BL6J mouse peritoneal macrophage-derived foam cells	12.5, 25, 50 μg/mL incubation for 24 h	/	ABCA1↑, ABCG1↑, CD36↓, SR-A1↓	Promote cholesterol efflux	[127]
**4**	Celosin II							
**5**	Maslinic acid	*Crataegus* *pinnatifida*	THP-1-derived foam cells	5, 10 μM incubation for 24 h	/	ABCA1↑, ABCG1↑, SR-A1↓, CD36↓	Promote cholesterol efflux, inhibit cholesterol intake	[130]
**6**	Diosgenin	*Dioscorea* *polystachya*	THP-1 and C57/BL6J mouse peritoneal macrophage-derived foam cells	10, 20, 40, 80 μM incubation for 24 h	miR-19b	ABCA1↑, miR-19b↓	Promote cholesterol efflux	[132]
**7**	*Panax* *notoginseng* saponins	*Panax* *notoginseng*	THP-1-derived foam cells	25, 50, 100 mg/L incubation for 12 h	/	LXRα↑, ABCA1↑, ABCG1↑, NF-κB↓	Promote cholesterol efflux	[135]

“↑” indicates an upward adjustment, “↓” indicates downward adjustment.

### 4.3. Diterpenoids

**Figure 6:**
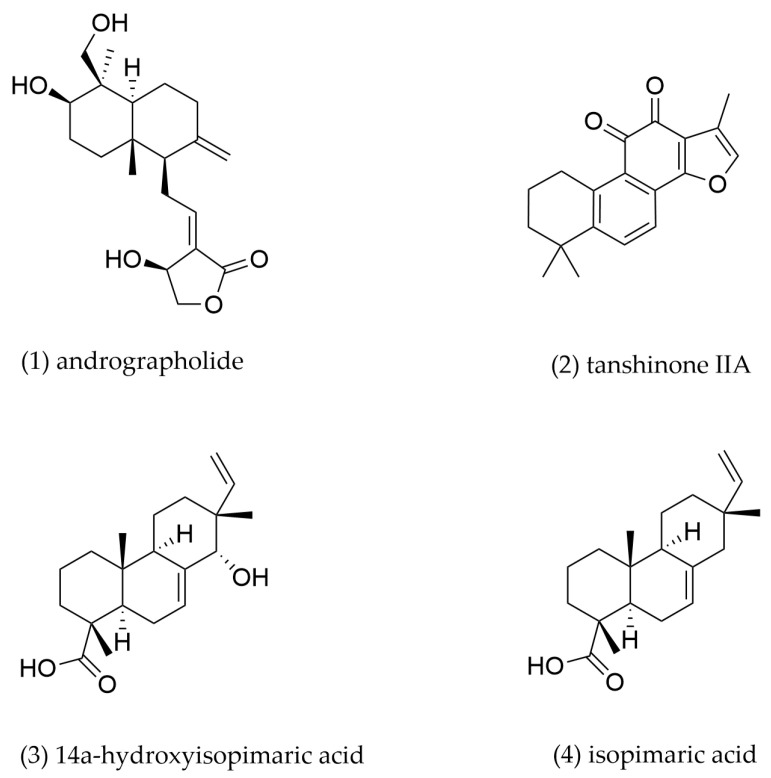
Structure of diterpenoid.

Diterpenoids, a group of 20 carbon atoms composed of four isoprene units, are widely distributed in plants and exhibit diverse pharmacological properties. Recent studies have highlighted their potential in modulating macrophage cholesterol metabolism. Major diterpenoids from TCMs and their underlying mechanisms are presented and summarized in Figure 6 and Table 3.

Andrographolide, a diterpenoid compound derived from *Andrographis paniculata*, exhibits diverse pharmacological properties, including anti-inflammatory, anticancer, anti-obesity, antidiabetic, and cardiovascular protective effects [136]. Research by Wu et al. revealed that andrographolide treatment (2.5 mg/kg) significantly reduced aortic plaque accumulation in *ApoE^−/−^* mice [137]. Similarly, andrographolide treatment (40 mg/kg) markedly decreased aortic foam cell formation in a rat model [138]. In vitro studies further revealed that andrographolide (0.5, 1 μM) inhibited ox-LDL-induced foam cell formation in J774A.1 macrophages by activating LXRα, which upregulated ABCA1 and ABCG1 expression while downregulating CD36 [139]. However, andrographolide treatment has no effect on altering SR-A1 expression.

Tanshinone IIA, a major bioactive constituent from *Salvia miltiorrhiza* [140], exhibits significant anti-atherosclerotic activity. Liu et al. demonstrated that Tanshinone IIA reduced macrophage infiltration and attenuated the atherosclerotic plaque formation in *ApoE^−/^^−^* mice. Mechanistically, Tanshinone IIA markedly reduced the SR-A1 expression and increased ABCA1 and ABCG1 expression in lipid-laden macrophages through activating the extracellular signal-regulated kinase (ERK)/nuclear factor erythroid 2-related factor 2 (Nrf2)/HO-1 pathway [141]. Tan et al. found that Tanshinone IIA promoted cholesterol efflux and decreased cellular lipid content in THP-1 macrophages through upregulating Omentin-1 and ABCA1 expressions. These beneficial effects by Tanshinone IIA were blocked by knockdown of Omentin-1, indicating that these effects were most likely achieved via the Omentin-1/ABCA1 pathway [142].

The diterpenoids from the aerial parts of *Callicarpa rubella* exhibited a potential inhibitory effect on ox-LDL-induced macrophage foam cell formation, which suggests that these compounds may be promising candidates in the treatment of AS [143]. Two diterpenoids, 14α-hydroxyisopimaric acid and isopimaric acid, were isolated by Zhang et al. from *Callicarpa rubella* and demonstrated potential anti-atherosclerotic activities [144]. These two diterpenoids significantly attenuated ox-LDL-induced RAW264.7-derived foam cell formation in macrophages through activation of the PPARγ-LXRα signaling pathway, which subsequently upregulated ABCA1 and ABCG1 expression.

**Table 3:** The mechanism of action of diterpenoids.

Compound No.	Components	Source TCM	Experimental Model	Dosage	Pathway	Mechanism	Pharmacological Effects	Ref.
**1**	Andrographolide	*Andrographis paniculata*	J774A.1-derived foam cells	0.5, 1 μM incubation for 24 h	LXRα	LXRα↑, ABCA1↑, ABCG1↑, CD36↓	Promote cholesterol efflux, inhibit cholesterol intake	[139]
**2**	Tanshinone IIA	*Salvia* *miltiorrhiza*	THP-1-derived foam cells	1, 3, 10 μM incubation for 24 h	LXRα/Nrf2/HO-1	SR-A1↓, ABCA1↑, ABCG1↑	Promote cholesterol efflux, inhibit cholesterol intake	[141]
			THP-1-derived foam cells	20, 40, 80 mg/L incubation for 24 h	Omentin-1/ABCA1	ABCA1↑, Omentin-1↑	Promote cholesterol efflux	[142]
**3**	14α-hydroxyisopimaric acid	*Callicarpa* *rubella*	RAW264.7-derived foam cells	15 μM incubation for 24 h	PPARγ/LXRα	PPARγ↑, LXRα↑, ABCA1↑, ABCG1↑	Promote cholesterol efflux	[144]
**4**	Isopimaric acid	*Callicarpa* *rubella*	RAW264.7-derived foam cells	15 μM incubation for 24 h	PPARγ/LXRα	PPARγ↑, LXRα↑, ABCA1↑, ABCG1↑	Promote cholesterol efflux	[144]

“↑” indicates an upward adjustment, “↓” indicates downward adjustment.

### 4.4. Alkaloids

**Figure 7:**
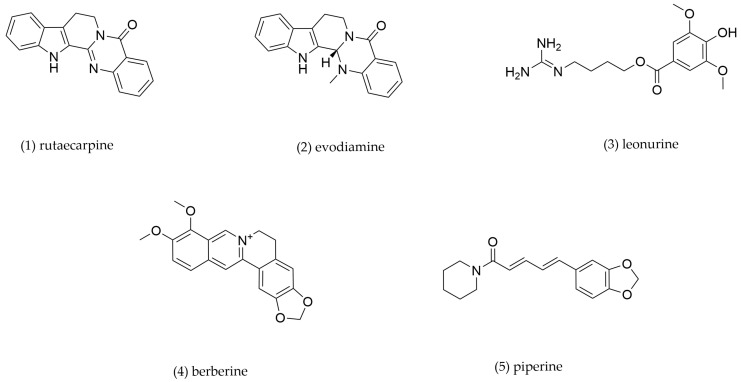
Structure of alkaloids.

Alkaloids, a diverse class of nitrogen-containing secondary metabolites, exhibit numerous pharmacological activities. Recent studies have demonstrated their protective effects against AS through multiple mechanisms. Major alkaloids from TCMs and their underlying mechanisms are presented and summarized in Figure 7 and Table 4.

Rutaecarpine and evodiamine, bioactive alkaloids derived from *Evodia rutaecarpa*, have emerged as promising therapeutic candidates for metabolic disorders due to their lipid-modulating ability. Xu et al. revealed that rutaecarpine upregulated the expression of cholesterol transporters (ABCA1, ABCG1, and SR-B1) related to LXRα and LXRβ in RAW264.7 macrophages, thereby attenuating foam cell formation and exhibiting anti-atherosclerotic effects [145]. Wang et al. demonstrated that evodiamine directly binds to ABCA1, enhancing cholesterol efflux from THP-1-derived macrophages [146].

Leonurine, a bioactive alkaloid derived from *Leonurus japonicus* [147], exhibits potent anti-atherosclerotic activity through modulating cholesterol metabolism. Mechanistic studies demonstrated that leonurine activated the PPARγ/LXRα signaling pathway in THP-1-derived foam cells, resulting in significant upregulation of ABCA1 and ABCG1 expression to stimulate cholesterol efflux capacity and consequently suppress intracellular lipid accumulation. Furthermore, leonurine administration markedly attenuated atherosclerotic plaque formation in *ApoE^−/^^−^* mice [64].

Berberine, a principal bioactive quaternary ammonium alkaloid from *Coptis chinensis*, demonstrates significant anti-inflammatory and anti-atherosclerotic activities [148,149]. Experimental evidence reveals its multi-target mechanisms in AS. Berberine improved glucolipid metabolism in *ApoE^−/^^−^* mice with diabetic AS [150]. Berberine downregulated LOX-1 expression, inhibited SR-mediated lipid uptake, upregulated ABCA1, ABCG1, and SR-B1 expression to enhance cholesterol efflux in human macrophages [151]. These coordinated actions effectively regulate lipid homeostasis by suppressing AP-1 activity and activation of the Nrf2/HO-1 pathway, reducing cholesterol uptake and promoting efflux [152].

Piperine, a bioactive alkaloid isolated from *Piper nigrum* [153], exhibits diverse pharmacological properties, including anti-inflammatory, antiangiogenesis, antioxidant, antidiabetic, antiobesity, cardioprotective, and antimicrobial activities [154]. Recent research by Wang et al. demonstrated that the potential atheroprotective effect of piperine is achieved through dose-dependent upregulation of ABCA1 in THP-1-derived macrophages [155].

**Table 4:** The mechanism of action of alkaloids.

Compound No.	Components	Source TCM	Experimental Model	Dosage	Pathway	Mechanism	Pharmacological Effects	Ref.
**1**	Rutaecarpine	*Evodia rutaecarpa*	RAW264.7-derived foam cells	0.035, 0.35, 3.48, 34.80 μM incubation for 24 h	LXRα/LXRβ	ABCA1↑, ABCG1↑, SR-B1↑	Promote cholesterol efflux	[145]
**2**	Evodiamine		THP-1-derived foam cells	1, 3, 10, 20 μM incubation for 24 h	/	ABCA1↑	Promote cholesterol efflux	[146]
**3**	Leonurine	*Leonurus* *japonicu*	THP-1-derived foam cells	5, 10, 20, 40, 80 μM incubation for 24 h	PPARγ/LXRα	PPARγ↑, LXRα↑, ABCA1↑, ABCG1↑	Promote cholesterol efflux	[64]
**4**	Berberine	*Coptis chinensis*	THP-1-derived foam cells, C57/BL6J mouse peritoneal macrophage-derived foam cells	1, 3, 10 μM incubation for 24 h	Nrf2/HO-1	ABCA1↑, ABCG1↑, SR-B1↑, LOX-1↓, SR-A1↓	Promote cholesterol efflux, inhibit cholesterol intake	[152]
**5**	Piperine	*Piper nigrum*	THP-1-derived foam cells	25, 50, 100 μM incubation for 24 h	/	ABCA1↑	Promote cholesterol efflux	[155]

“↑” indicates an upward adjustment, “↓” indicates downward adjustment.

### 4.5. Polysaccharide

Polysaccharides are high-molecular-weight polymers that serve as essential structural components in TCMs. They exhibit diverse pharmacological properties, including hypolipidemic, anti-inflammatory, and antioxidant ones. Polysaccharides have emerged as promising therapeutic agents for the management of CVDs. The mechanisms of action of representative polysaccharides are presented in Table 5.

The polysaccharides derived from *Pleurotus eryngii* exhibit multiple pharmacological effects, including appetite stimulation, spleen fortification, digestion promotion, and hypolipidemic/hypotensive activities [156]. Mechanistic studies by Nakahara et al. [157] revealed that these substrate polysaccharides significantly reduced cellular and murine lipid levels. The anti-atherogenic effect is mediated through the downregulation of CD36 by *Pleurotus eryngii* polysaccharide, which consequently inhibits macrophage cholesterol uptake [158,159].

Fucoidan, a sulfated polysaccharide predominantly extracted from *Laminaria japonica*, is characterized by its fucose-rich composition and sulfate ester groups [160]. It exhibits potent antithrombotic and antioxidant properties. Experimental studies have demonstrated its significant anti-atherosclerotic effects through multiple mechanisms. Fucoidan could attenuate atherosclerotic plaque formation in *ApoE^−/^^−^* mouse models [161], upregulate ABCA1 expression in THP-1 macrophages via LXR-α activation [162], enhance ox-LDL-induced RCT, and inhibit SR-A1 expression in THP-1 macrophages [163]. These coordinated actions collectively suppress foam cell formation and retard atherosclerotic progression.

*Opuntia dillenii* Haw polysaccharide (ODP-Ia), a principal bioactive component extracted from *Opuntia dillenii*, demonstrates significant anti-atherosclerotic properties. Mechanistic studies revealed [164] that ODP-Ia enhanced RCT through activation of the PPARγ-LXRα signaling pathway, resulting in upregulated expression of cholesterol transporters ABCA1, ABCG1, and SR-B1 in THP-1 macrophages.

**Table 5:** The mechanism of action of polysaccharides.

Compound No.	Components	Source TCM	Experimental Model	Dosage	Pathway	Mechanism	Pharmacological Effects	Ref.
**1**	*Pleurotus eryngii* polysaccharide	*Pleurotus* *eryngii*	RAW264.7-derived foam cells	5, 100, 200 μg/mL incubation for 24 h	/	CD36↓	Inhibit cholesterol intake	[158]
**2**	Fucoidan	*Laminaria* *japonica*	THP-1-derived foam cells	50 μg/mL incubation for 24 h	LXRα	LXRα↑, ABCA1↑, SR-A1↓	Promote cholesterol efflux	[162,163]
**3**	*Opuntia dillenii* Haw polysaccharide	*Opuntia* *dillenii*	THP-1-derived foam cells	5, 10, 20 nM incubation for 24 h	PPARγ/LXRα	PPARγ↑, LXRα↑, ABCA1↑, ABCG1↑, SR-B1↑	Promote cholesterol efflux	[164]

“↑” indicates an upward adjustment, “↓” indicates downward adjustment.

### 4.6. Other Compounds

**Figure 8:**
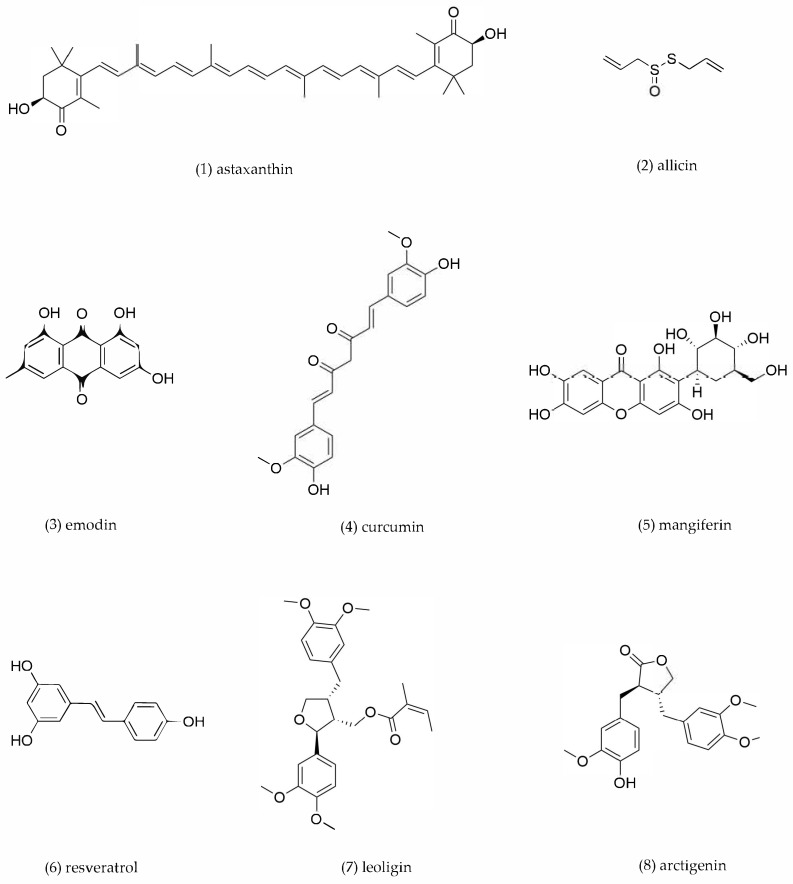
Structure of other compounds.

Other types of compounds from TCMs, for example, carotenoid, lignan, and anthraquinone, also modulate macrophage cholesterol metabolism to exert anti-atherosclerotic effects. The mechanisms of action of representative compounds (Figure 8) are presented in Table 6.

Astaxanthin, a potent xanthophyll carotenoid widely distributed in *Haematococcus pluvialis*, exhibits exceptional antioxidant capacity owing to its unique molecular structure. These properties confer significant biomedical potential, particularly in anti-inflammatory and immunomodulatory applications [165]. Zhang et al. demonstrated that astaxanthin enhanced ABCA1-mediated cholesterol efflux and protected against ox-LDL-induced cytotoxicity in RAW264.7 macrophages. Astaxanthin treatment suppressed miR-3073b-5p expression and upregulated circTPP2 and ABCA1 expression, thus promoting cholesterol efflux and enhancing the ability of RCT [166].

Allicin, an organosulfur compound derived from *Allium sativum*, has shown beneficial effects on several cardiovascular risk factors [167]. Mechanistic studies demonstrated that allicin activated the PPARγ/LXRα signaling pathway to induce upregulation of ABCA1 in THP-1-derived foam cells, thereby promoting cholesterol efflux and reducing lipid accumulation [168].

Emodin, an anthraquinone derivative extracted from *Rheum palmatum*, demonstrates potential anti-atherosclerotic effects. Fu et al. [169] investigated emodin’s mechanism of action by treating oxLDL-induced THP-1 macrophages with varying concentrations of emodin (0–10 μM) for 18 h. Western blot and RT-PCR analyses revealed that emodin treatment significantly upregulated the expression of key cholesterol efflux regulators, including LXR-α, ABCA1, ABCG1, and PPARγ. These enhanced expressions promoted cholesterol efflux, consequently reducing foam cell formation. Moreover, emodin promoted apoA-1-mediated cholesterol efflux, which was significantly abolished by pretreatment with the PPAR-γ selective antagonist or specific small interfering RNA for PPAR-γ. These findings suggest that emodin exerts its anti-atherosclerotic effects through the PPARγ/LXR-α/ABCA1/ABCG1 signaling pathway.

Curcumin, a principal bioactive polyphenol derived from *Curcuma longa*, exhibits diverse pharmacological properties including potent anti-inflammatory and anti-tumor activities [170]. Experimental evidence has demonstrated that curcumin supplementation significantly downregulated TLR4 expression in *ApoE^−/^^−^* mice [171] while attenuating cholesterol absorption and atherosclerotic plaque formation [172]. Intravenous administration of curcumin effectively reduced serum lipid levels and inhibited AS progression in rabbit models [173]. A mechanistic study by Zhong et al. revealed that curcumin significantly upregulated HO-1, ABCA1, and SR-B1 expression and increased Nrf2-driven luciferase activity in RAW264.7 macrophage. The increased SR-B1 and ABCA1 expression induced by curcumin was partly abolished by blocking HO-1, which was inhibited by Nrf2 siRNA. It is suggested that curcumin treatment activates the Nrf2-ARE signaling pathway and upregulates HO-1 expression, which mediates SR-B1 and ABCA1 expression, thereby increasing cholesterol efflux [174].

Mangiferin, a kind of polyphenol chemical compound separated from *Mangifera indica* leaves, has anti-inflammatory, anti-virus, and hypoglycemic and hypolipidemic properties [175,176]. Recent studies have demonstrated its therapeutic potential in lipid metabolism regulation. In vivo, mangiferin-loaded nanoparticles effectively reduced hyperlipidemia in rat models [177]. In *ApoE^−/−^* mice, mangiferin administration significantly attenuated atherosclerotic plaque formation. Mechanistically, mangiferin upregulated LXRα, PPARγ, and ABCA1/G1 expression in RAW264.7 macrophages, thus enhancing cholesterol efflux and suppressing lipid accumulation [178]. Chen et al. found that combining mangiferin with the LXRα agonist T0901317 synergistically amplified cholesterol efflux, further supporting its role in macrophage RCT. Collectively, these findings indicate that mangiferin exerts anti-atherogenic effects primarily via the PPARγ-LXRα-ABCA1/G1 pathway [179].

Resveratrol, a polyphenolic compound abundant in *Reynoutria japonica*, exhibits potent cholesterol-modulating properties [180]. Ye et al. found that resveratrol enhanced ABCA1- and ABCG1-mediated cholesterol efflux to attenuate oleate-induced lipid accumulation in RAW264.7 macrophages via PPARα/γ activation. These attenuating effects were observed in synthetic PPAR agonist treatment (e.g., WY14643 and pioglitazone), which similarly promoted RCT and reduced intracellular lipid deposition via analogous pathways. These findings support the effect of resveratrol by activating PPARα/γ signaling [181].

Leoligin, a bioactive lignan derived from *Leontopodium leontopodioides*, demonstrates significant atheroprotective potential through modulation of macrophage cholesterol homeostasis. Wang et al. revealed that leoligin upregulated ABCA1 and ABCG1 expression in THP-1-derived macrophages, thereby enhancing cholesterol efflux capacity [182].

Arctigenin, a principal bioactive compound derived from *Arctium lappa* [183], demonstrates atheroprotective properties through modulation of RCT. Xu et al. demonstrated that arctigenin dose-dependently enhanced cholesterol efflux in oxLDL-loaded THP-1 macrophages through the activation of PPARγ/LXR-α signaling and subsequent upregulation of ABCA1 and ABCG1 transporters [184].

**Table 6:** The mechanism of action of other compounds.

Compound No.	Components	Source TCM	Experimental Model	Dosage	Pathway	Mechanism	Pharmacological Effects	Ref.
**1**	Astaxanthin	*Haematococcus pluvialis*	RAW264.7-derived foam cells	0.5, 5, 50 μM incubation for 48 h	circTPP2/miR-3073b-5p/ABCA1	ABCA1↑, circTPP2↑, miR-3073b-5p↓	Promote cholesterol efflux	[166]
**2**	Allicin	*Allium* *sativum*	THP-1-derived foam cells	2.5. 5, 10, 20, 40 mg/mL incubation for 24 h	PPARγ/LxRα	PPARγ↑, LXRα↑, ABCA1↑	Promote cholesterol efflux	[168]
**3**	Emodin	*Rheum* *palmatum*	THP-1-derived foam cells	0–10 μM incubation for 18 h	PPARγ/LXRα	PPARγ↑, LXRα↑, ABCA1↑, ABCG1↑	Promote cholesterol efflux	[169]
4	Curcumin	*Curcuma* *longa*	RAW264.7-derived foam cells	10, 20, 40 μM incubation for 12 h	Nrf2/ARE	HO-1↑, ABCA1↑, SR-B1↑	Promote cholesterol efflux	[174]
**5**	Mangiferin	*Mangifera indica*	RAW264.7-derived foam cells	5, 10, 20 μM incubation for 24 h	PPARγ/LXRα-ABCA1/ABCG1	PPARγ↑, LXRα↑, ABCA1↑, ABCG1↑	Promote cholesterol efflux	[179]
**6**	Resveratrol	*Reynoutria japonica*	RAW264.7-derived foam cells	1.5 μg/mL incubation for 24 h	PPARα/γ	PPARγ↑, PPARα↑, ABCA1↑, ABCG1↑	Promote cholesterol efflux	[181]
**7**	Leoligin	*Leontopodium leontopodioides*	THP-1-derived foam cells	10, 20, 40 μM incubation for 24 h	/	ABCA1↑, ABCG1↑	Promote cholesterol efflux	[182]
**8**	Arctigenin	*Arctium lappa*	THP-1-derived foam cells	10, 50, 100 μM incubation for12 h	PPARγ/LXRα	PPARγ↑, LXRα↑, ABCA1↑, ABCG1↑	Promote cholesterol efflux	[184]

“↑” indicates an upward adjustment, “↓” indicates downward adjustment.

## 5. Conclusions and Outlook

AS, a leading underlying cause of many CVDs, is increasingly prevalent in the population [5]. Dysregulated cholesterol metabolism in macrophages leads to abnormal cholesterol accumulation, which not only promotes foam cell formation but also triggers systemic inflammatory responses [185], exacerbating the progression of AS.

TCM, refined through millennia of clinical practice, has gained increasing international recognition. Increasing evidence has demonstrated that TCMs, TCM formulas and their related compounds regulate cholesterol homeostasis to attenuate the AS progress. However, comprehensive reviews specifically addressing macrophage cholesterol metabolism and TCM compounds targeting macrophage cholesterol metabolism for AS management remain scarce.

In this review, current research on macrophage cholesterol metabolism in AS pathogenesis and the therapeutic potential of TCM bioactive compounds is systematically summarized. Four processes, including cholesterol uptake, efflux, and cholesterol esterification and hydrolysis, are involved in macrophage cholesterol metabolism. Multiple key regulators of macrophage cholesterol metabolism involved in these four steps, such as SR-A1, CD36, ABCA1, ABCG1, and SR-B1, are potential therapeutic targets for treating AS. Various TCM-derived bioactive components, such as flavonoids, saponins, terpenoids, polysaccharides, and alkaloids, regulate cholesterol homeostasis through multiple pathways. Their mechanisms involve cholesterol efflux enhancement (upregulation of ABCA1, ABCG1, and SR-B1 expression), cholesterol uptake inhibition (downregulation of CD36 and SR-A1), and modulation of signaling pathways (PPARγ/LXRα, AP-1/HO-1, LXRα/NF-κB, and TLR4/HO-1). The PPARγ/LXRα signaling pathway plays a critical role in atherosclerosis pathogenesis by modulating macrophage cholesterol efflux and inflammatory responses. Additionally, the AP-1/HO-1, LXRα/NF-κB, and TLR4/HO-1 signaling pathways influence disease progression by regulating inflammatory mechanisms. These coordinated actions attenuate foam cell formation and exert anti-AS effects.

Although many TCMs’ compounds have presented potential anti-AS effects, the research about TCMs’ compounds in regulating cholesterol homeostasis is still needed to be further explored. Given the differences between preclinical findings and clinical applications [186], there are a few limitations in this review. Some sections rely on a small number of studies, and most of the reviewed compounds were investigated in vitro. Only 11 of the reviewed compounds, accounting for less than 50%, were evaluated both in vitro and in vivo in cholesterol metabolism in macrophages. All these 11 compounds showed efficacy in vitro and in vivo. There is a limited number of these reviewed compounds that have stepped into clinical trials. For example, Tanshinone IIA, the pharmacokinetic study of which in humans has been finished in China (Registration Number: CTR20191455). Based on data from 2009–2021, TCM components have been less well studied in clinical trials, with a total of 97 TCM compounds accounting for 9.7% of the total number of platform-registered clinical trials of drugs for cardiovascular-metabolic diseases [187]. This is possibly due to the differences between the unhuman model and humans. The in vitro and in vivo models cannot fully imitate the environment in humans. The most frequently used THP-derived foam cells do not have the coexistence of M1 and M2 in humans. Two classic transgenic mouse models, *apoE^−/−^* and *Ldlr^−/−^*, constitute the primary platforms for studying AS. However, there exist crucial differences between mice and humans, such as the unhumanized lipoprotein profile, and the different plaque progression and characteristics [188]. The effect of the metabolites cannot be evaluated in vitro. Second, among our reviewed compounds, some have been used in clinics for treating other diseases, such as berberine as an antimicrobial drug, and piperine for treating Seizures in China. These approved drugs for treating other diseases indicate their safety in AS treatment. However, there are still a number of reviewed compounds that have not been evaluated for their safety in vivo by the AS model. Third, the metabolites of most anti-AS TCM compounds in vivo are less investigated. It is still unknown whether these compounds act in their original form or in their transforming form in vivo. In addition, little research has been conducted on the structure-activity relationship of these reviewed anti-AS compounds. For example, alkaloids rutaecarpine and evodiamine, diterpenoids 14α-hydroxyisopimaric acid and isopimaric acid, flavonoids kaempferol and quercetin, they share the same scaffold but have different substituents. There is no research on the relationship between their structures and their anti-AS activities. Therefore, there is a great challenge for translating potential anti-AS TCM compounds from preclinical to clinical research.

Through comprehensive analysis of their anti-AS mechanisms, we aimed to provide novel insights for AS research strategies. To optimize TCM resource utilization, we propose the following research directions:

(1) Identification of potential anti-AS compounds of TCMs and TCM formulas

The anti-AS compounds of many TCMs and TCM formulas remain unknown. Using the convenient UPLC-Q-TOF-MS/MS technique [189], we can explore the chemical composition of TCMs. This technique allows for the identification of bioactive compounds in TCM extracts and formulas that interact with key regulators in macrophage cholesterol metabolism before conducting in vitro and in vivo pharmacological evaluations.

(2) Structure–activity relationship research

Applying synthesis technology, nanotechnology, and virtual computer-aided drug design to modify TCM compounds to generate a serial of generate a series of Chinese medicine derivatives with improved bioavailability, such as CREB-inhibiting HPDA/Zn nanoparticles, demonstrate potential for stabilizing atherosclerotic plaques by modulating macrophage inflammatory responses, offering a complementary approach to TCM-derived compounds [190]. The key scaffold for anti-AS activities is potentially indicated further through investigating their influences on pharmacological activity and toxicity in vitro or in vivo.

(3) Pharmacokinetics research

To employ advanced techniques, such as in vivo/in vitro metabolic simulation and isotope tracing methodologies, to identify bioactive metabolite forms of TCM compounds and their pharmacokinetic properties, absorption, distribution, metabolism, and excretion. Based on the PK parameters (e.g., metabolic stability, half-life), potential lead compounds will be optimized to improve their druggability. Additionally, epigenetic regulation [191] and engineered macrophage therapies [192] represent another layer of intervention in macrophage cholesterol metabolism.

(4) Interdisciplinary integration to identify targets

Combining cutting-edge technologies from biology (e.g., affinity purification, activity-based probes, and gene reduction or overexpression), and computational science (e.g., computational modeling of compound-receptor interactions), for example, integrating molecular docking, network pharmacology, and transcriptome to investigate the interactions of TCM compounds [193] and key regulators in regulating cholesterol homeostasis to identify the main target. Emerging tools like network toxicology and machine learning can refine target prediction for TCM compounds, bridging gaps between traditional pharmacology and modern systems biology [194].

(5) Synergistic effects of TCM bioactive compounds

TCM, characterized by multi-constituents and multi-targets, exerts anti-AS effects possibly through different compounds on different targets. Astragaloside IV, another TCM bioactive, attenuates atherosclerosis via the PI3K/Akt/mTOR pathway activation, reducing oxidative stress and improving lipid metabolism in *apoE^−/−^* models, further underscoring the multi-target potential of TCM [195]. Exploring the synergistic effects of TCM bioactive compounds will be useful for their use in combination therapies in the future.

These investigations will establish a foundation for developing innovative anti-AS therapies that harness the multi-target pharmacological advantages of TCM, ultimately enhancing their clinical translation potential.

## Data Availability

No new data were created or analyzed in this study. Data sharing is not applicable.

## Abbreviations

The following abbreviations are used in this manuscript:

ABCA1	ATP-binding cassette transporter A1
ABCG1	ATP-binding cassette transporter G1
ACAT1	cholesterol acyltransferase 1
acLDL	acetylated LDL
apoA-1	apolipoprotein A-1
AS	Atherosclerosis
CD36	cluster of differentiation 36
CEs	cholesteryl esters
CVDs	Cardiovascular diseases
ECs	endothelial cells
ERK	extracellular signal-regulated kinase
FASN	fatty acid synthase
FC	free cholesterol
HDAC9	histone deacetylase 9
HDL	high-density lipoprotein
HMG-CoA	3-hydroxy-3-methyl glutaryl coenzyme A reductase
HO-1	heme oxygenase-1
ICAMs	intercellular cell adhesion molecules
IFN-γ	interferon-γ
IL-17A	interleukins 17A
IL-33	interleukins 33
LDLRs	low-density lipoprotein receptors
LOX-1	lectin-like oxidized low-density lipoprotein receptor-1
LXR	Liver X receptor
MAPK	mitogen-activated protein kinase
M-CSF	macrophage colony-stimulating factor
mmLDL	minimally oxidized LDL
nCEH	neutral cholesteryl ester hydrolase
Nrf2	nuclear factor erythroid 2-related factor 2
ODP-Ia	*Opuntia dillenii* Haw polysaccharide
ox-LDL	oxidized low-density lipoprotein
PMA	phorbol 12-myristate 13-acetate
PON1	paraoxonase-1
PPAR	peroxisome proliferator-activated receptor
RCT	reverse cholesterol transport
SOD	superoxide dismutase
SR	scavenger receptors
SR-A1	scavenger receptor class A1
SR-B1	scavenger receptor class B type 1
SYK	spleen tyrosine kinase
TCM	traditional Chinese medicine
TGF-β	transforming growth factor-β
TLR4	toll-like receptor 4
VCAMs	vascular cell adhesion molecules
VLDL	very low-density lipoprotein
